# Bioassay-Guided Different Extraction Techniques of *Carica papaya* (Linn.) Leaves on In Vitro Wound-Healing Activities

**DOI:** 10.3390/molecules25030517

**Published:** 2020-01-24

**Authors:** Husnul Hanani Soib, Hassan Fahmi Ismail, Fitrien Husin, Mohamad Hafizi Abu Bakar, Harisun Yaakob, Mohamad Roji Sarmidi

**Affiliations:** 1 Department of Bioprocess and Polymer Engineering, School of Chemical and Energy Engineering, Faculty of Engineering, University Teknologi Malaysia, Skudai 81310, Malaysia; hannanmh88@gmail.com; 2 Institute of Marine Biotechnology, Universiti Malaysia Terengganu, Kuala Terengganu 21030, Malaysia; h.fahmi@umt.edu.my; 3 Institute of Bioproduct Development, University Teknologi Malaysia, Skudai 81310, Malaysia; fitrienh@gmail.com; 4 Bioprocess Technology Division, School of Industrial Technology, Universiti Sains Malaysia, Gelugor 11800, Penang, Malaysia; mhafizi88@usm.my; 5 Innovation Centre in Agritechnology for Advanced Bioprocessing (ICA), University Teknologi Malaysia, Skudai 81310, Malaysia; mroji@utm.my

**Keywords:** *Carica papaya*, antioxidant, wound healing, extraction, conventional

## Abstract

Herbal plants are traditionally utilized to treat various illnesses. They contain phytochemicals that can be extracted using conventional methods such as maceration, soxhlet, and boiling, as well as non-conventional methods including ultrasonic, microwave, and others. *Carica papaya* leaves have been used for the treatment of dengue, fungal, and bacterial infections as well as an ingredient in anti-aging products. Phytochemicals analysis detected the presence of kaempferol, myricetin, carpaine, pseudocarpaine, dehydrocarpaine I and II, ferulic acid, caffeic acid, chlorogenic acid, β-carotene, lycopene, and anthraquinones glycoside. Conventional preparation by boiling and simple maceration is practical, simple, and safe; however, only polar phytochemicals are extracted. The present study aims to investigate the effects of three different non-conventional extraction techniques (ultrasonic-assisted extraction, reflux, and agitation) on *C. papaya* phytochemical constituents, the antioxidant capacity, and wound-healing activities. Among the three techniques, the reflux technique produced the highest extraction yield (17.86%) with the presence of saponins, flavonoids, coumarins, alkaloids, and phenolic metabolites. The reflux technique also produced the highest 2, 2-diphenyl-1-picrylhydrazyl (DPPH) radical scavenging with an IC_50_ value of 0.236 mg/mL followed by ultrasonic-assisted extraction (UAE) (IC_50_: 0.377 mg/mL) and agitation (IC_50_: 0.404 mg/mL). At tested concentrations (3.125 µg/mL to 500 µg/mL), all extracts do not exhibit a cytotoxicity effect on the human skin fibroblast, HSF1184. Interestingly, reflux and UAE were active fibroblast proliferators that support 85% (12.5 µg/mL) and 41% (6.25 µg/mL) better cell growth, respectively. Additionally, during the early 24 h of the scratch assay, the migration rate at 12.5 µg/mL was faster for all extracts with 51.8% (reflux), 49.3% (agitation), and 42.5% (UAE) as compared to control (21.87%). At 48 h, proliferated cells covered 78.7% of the scratch area for reflux extract, 63.1% for UAE, 61% for agitation, and 42.6% for control. Additionally, the collagen synthesis was enhanced for 31.6% and 65% after 24 and 48 h of treatment for reflux. An HPLC-MS/MS-QTOF (quadruple time-of-flight) analysis of reflux identified nine phytochemicals, including carpaine, kaempferol 3-(2G-glucosylrutinoside), kaempferol 3-(2″-rhamnosylgalactoside), 7-rhamnoside, kaempferol 3-rhamnosyl-(1->2)-galactoside-7-rhamnoside, luteolin 7-galactosyl-(1->6)-galactoside, orientin 7-O-rhamnoside, 11-hydroperoxy-12,13-epoxy-9-octadecenoic acid, palmitic amide, and 2-hexaprenyl-6-methoxyphenol. The results suggested that reflux was the best technique as compared to ultrasonic and agitation.

## 1. Introduction

Skin is a major organ comprising 15%−20% of total body weight [1]. Microbial infection, ultraviolet light, chemical damages, and physical damages cause wounds that disrupt the structure and functionality of skin. Wound healing is a complex and dynamic process comprising multiple interactions among cellular structures, tissue layers, and different types of cells. It involves three major processes: inflammation, proliferation, and tissue remodeling. Upon skin injury, the body will undergo a homeostasis of vasoconstriction and platelet aggregation for blood clotting. Simultaneously, cells at the affected area secrete cytokines, water, salts, and proteins. Proliferation cascades are achieved by the migration of fibroblasts and keratinocytes along with the fibrin network, angiogenesis, and reepithelialization. During the final stage of tissue remodeling, type III collagen that is synthesized during the proliferation stage is remodeled into stronger type I collagen, which reduces scar size and increases the tensile strength. 

*Carica papaya* Linn, also known as betik in Malaysia, belongs to the *Caricaceae* family. It is a soft-stemmed perennial plant that can grow up to 20 m without branches. It is the most cultivated plant in tropical and subtropical countries. In Malaysia, there are several varieties of *C. papaya* such as Eksotika, Sekaki, Subang, and Solo. However, among these varieties, Eksotika contained an abundance of metabolites, and it is the most popular variety in Malaysia. Studies revealed that the presence of compounds in the *C. papaya* exhibits various biological activities such as anti-diabetes [2], anti-fertility [3], anti-fungal [4,5], anti-bacterial [6,7], anti-tumor [8], antioxidant [9], anti-dengue [10], and anti-plasmodial [11], despite the different parts of the papaya plant. As described by Muhamad et al., 2017, Asghar et al., 2016, and Tay and Chong, 2016, the *C. papaya* plant contained an abundance of polar compounds rather than non-polar compounds [5,12,13]. Nugroho et al., 2017 reported seven flavonoid compounds from a butanol fraction of methanol extract of *C. papaya* leaves, including quercetin 3-(2^G^-rhamnosyrutinoside), kaempherol 3-(2^G^-rhamnosylrutinoside), quercetin 3-rutinoside, myricetin 3-rhamnoside, kaempherol 3-rutinoside, quercetin, and kaempherol, which have been tested for antioxidant activity by peroxynitrite scavenging assays [14]. The results showed that these flavonoids possessed potent antioxidant properties even when being compared to the positive control, which was *l*-penicillamine. Afzan et al., 2012 also has successfully detected malic acid, quinic acid, caffeoyl malate, quercetin-3-*O*-(2″,6″-di-*O*-rhamnopyranosyl) glucopyranoside (manghaslin), *p*-coumaroyl malate, kaempherol-3-*O*-(2″,6″-di-*O*-rhamnopyranosyl) glucopyranoside (clitorin), quercetin-3-*O*-rutinoside (rutin), kaempherol-3-*O*-rutinoside (nicotiflorin), and carpaine from leaves extract of *C. papaya* [15]. Furthermore, various studies on the effect of *C. papaya* on wound-healing activities have been previously reported [16,17,18,19]. The wound-healing properties were attributed by antioxidant, anti-inflammatory, and antimicrobial activities [20].

The demand for the natural product and plant-based phytochemicals has increased in trend by incorporating the herbs into modern medical practice. Realizing the impact of its use, a selective extraction technique has always been challenging. It is an important consideration to assure that the bioactive compounds that are biologically present in low concentrations from the plants remain preserved and yet are able to be used for the standardization of herbal products [21]. The phytochemical discovery from plant material can be achieved using an appropriate extraction process. Extraction is a process involving separating a mixture of compounds from non-miscible phases using a specific solvent. There are many factors influencing the quality (bioactivity) and quantity of the extract including types of solvents, techniques, and the parts of the plant used [22]. A proper extraction technique should be fully considered during extracting the bioactive constituents to assure that the target components are not lost, distorted, or destroyed. The emerging of modern extraction techniques by utilizing ultrasonic, supercritical fluid, microwave, and pressurized liquid proved to better extraction techniques with various advantages as compared to conventional methods (maceration, soxhlet, digestion, and reflux). For example, ultrasonic-assisted extraction (UAE) is easy to handle, has a faster extraction time, and consumes less energy [23]. 

*C. papaya* was commonly extracted by conventional techniques such as boiling [24,25] and shaking [26,27]. In this study, the ultrasonic, reflux, and agitation extraction techniques of *C. papaya* were analyzed for their effects on the secondary metabolites and phytochemicals profiling. Subsequently, the in vitro wound-healing capacity was examined using human skin fibroblasts. 

## 2. Results and Discussion

### 2.1. Percentage Yield and Secondary Metabolites Screening of *C. papaya* Extracts

The yield of the *C. papaya* extracts is presented in Table 1. The result showed that the reflux technique produced the highest yield of 17.86% followed by agitation (15.86%) and UAE (13.57%). The dissimilar result of the yield might due to the different mode of action in which the reflux method uses heat to break down the cell wall of the plant, while UAE and agitation use ultrasonic wave and mechanical force, respectively. The heat applied on the extract facilitates the diffusion and solubility of the bioactive compounds, resulting in an increase of releasing of intracellular compounds. At any non-conventional method applied, the efficiency of the extraction rate is dependent on the choice of solvent [28]. Moreover, the result also was in an agreement with the results reported by Sultana et al., 2009, who found that reflux produced the highest yield regardless of the plant material and solvent used [29].

Additionally, secondary metabolites screening showed the presence of saponins, flavonoids, coumarins, alkaloids, and phenolics in the leaves extracts of *C. papaya* by using all three extraction techniques (Table 2). However, terpenoids and steroids were not detected in the extracts. Various studies have shown that these compounds have a vast impact on wound-healing activities. For instance, saponin, which is known as ginsenoside from ginseng, has been proven to accelerate neovascularization in burn wound skin [30], while flavonoids also have been proven to increase the rate of wound contraction and hydroxyproline content, indicating an enhanced synthesis of collagen [31,32]. A triterpenoid saponin-rich fraction from *Centella asiatica* studied by Mahmood et al., 2016 can decrease Interleukin - 1β (IL-1β) and Nuclear factor kappa β (NF-κB), and it augments tissue regeneration and excision wound repair [33].

### 2.2. Antioxidant Activity by DPPH

**Figure 1:**
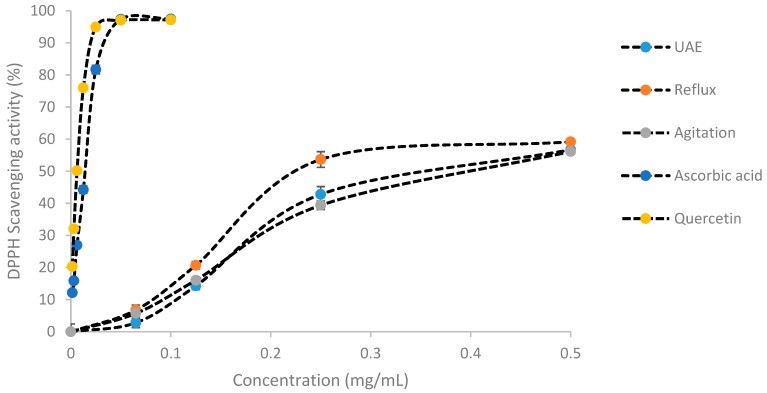
DPPH scavenging activity of UAE, reflux, and agitation *C. papaya* extracts. Results were expressed as the mean of three independent experiments.

The antioxidant capacity of *C. papaya* leaves extracts was measured via DPPH radical scavenging inhibitory activity as illustrated in Figure 1 and Table 3. The reflux technique produced the lowest IC_50_ (0.236 ± 0.009 mg/mL) followed by UAE (0.377 ± 0.014 mg/mL) and agitation (0.404 ± 0.009 mg/mL). Meanwhile, the IC_50_ values of the positive controls were much lower than the *C. papaya* extracts with 0.014 ± 0.002 mg/mL for ascorbic acid and 0.00625 ± 0.001 mg/mL for quercetin.

### 2.3. Wound-Healing Activities

#### 2.3.1. Cytotoxicity and Proliferation Activities

**Figure 2:**
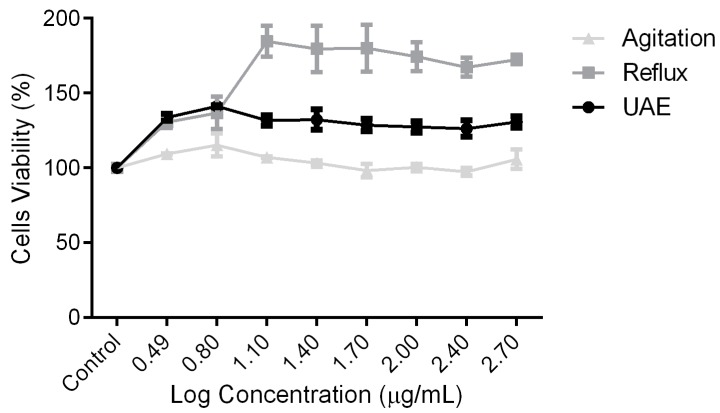
Cytotoxicity effect of *C. papaya* leaves extracts on the cell viability of HSF1184. (**A**) UAE, (**B**) reflux, (**C**) agitation. Results are expressed as the mean of three independent experiments.

Basic cytotoxicity and cell proliferation tests were carried out using a sulforhodamine staining assay. In this study, for all the tested concentrations (12.5 µg/mL to 500 µg/mL), no cytotoxicity effects were observed for all the extracts, as shown in Figure 2. Interestingly, extracts produced from UAE (Figure 2A) and reflux (Figure 2B) significantly proliferated the cells’ population. Moreover, reflux enhances the cells’ number up to 85% higher than control, suggesting the suitability of *C. papaya* extracts for wound-healing agents. 

#### 2.3.2. Migration Effects of *C. papaya* Extract on HSF1184 Cell Lines 

**Figure 3:**
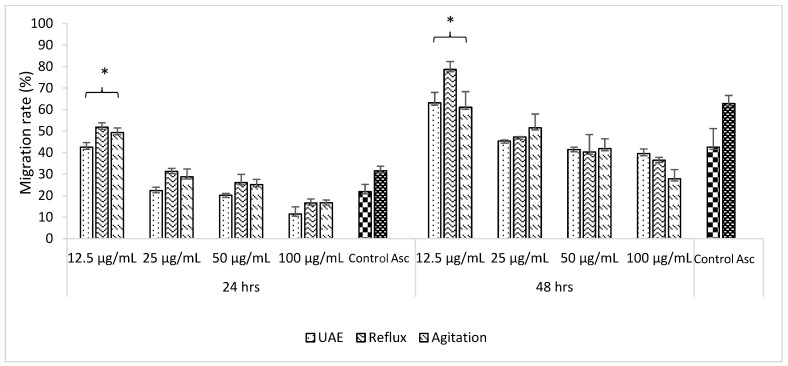
Effect of *C. papaya* extracts obtained from UAE, reflux, and agitation extraction techniques on the migration and proliferation rate of scratch HSF1184 cells. The migration rate was analyzed by using Image-J software. Results are expressed as the mean ± SEM of three independent experiments.

To determine the effect of *C. papaya* extracts on the cells’ migration and proliferation rate, the scratch areas on post-confluent HSF 1184 were treated for 48 h with non-toxic doses of extracts ranging from 12.5 to 100 µg/mL. Figure 3 showed that in the early 24 h of treatment, the migration rate was faster at lower concentrations as compared to higher concentrations for all extracts. At 12.5 µg/mL, as compared to control, the migration rate was 30% higher in reflux, followed by agitation (27.5%) and UAE (20.7%). Meanwhile, for other concentrations, no significant differences were measured in the early 24 h of treatment. Cell migration indicates the second phase of the wound-healing mechanism characterized by the migration of existing cells in the early 24 h post-wounded, followed by the proliferation of new cells on the wounded area [34]. 

**Figure 4:**
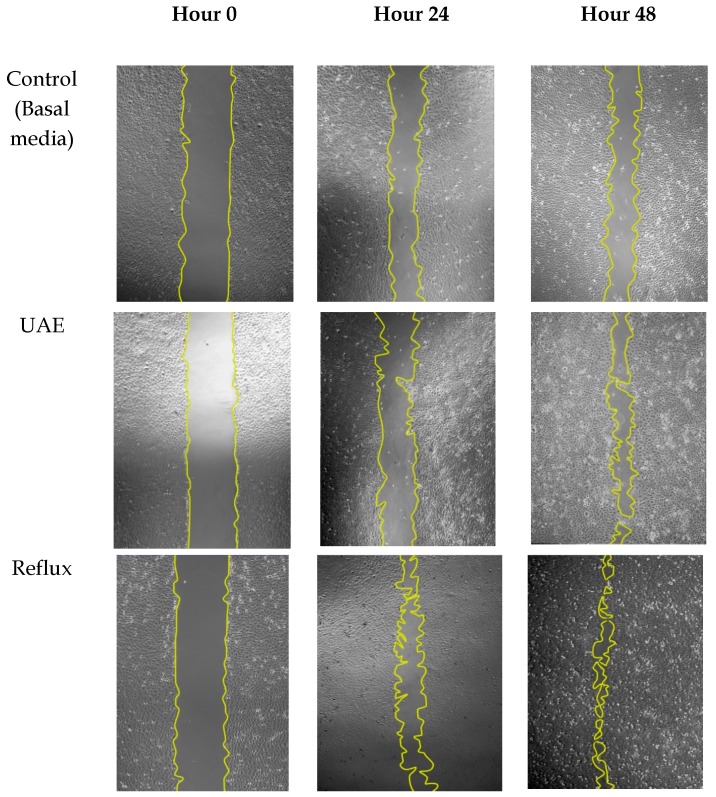

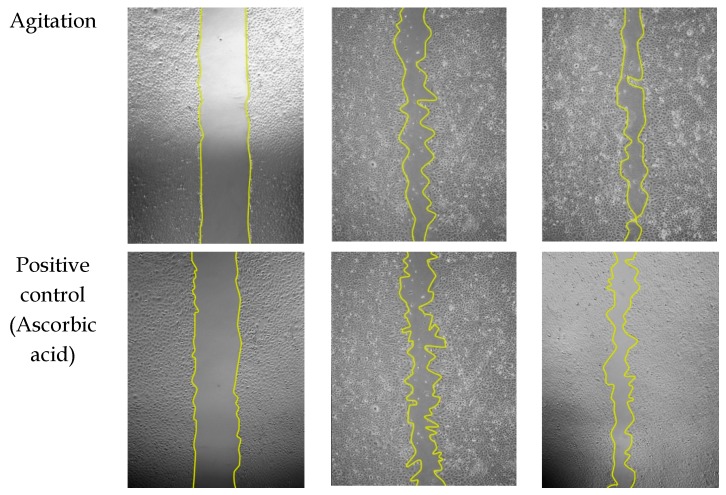
Images of the scratch area during the treatment of 12.5 µg/mL of extracts for 24 h and 48 h. The migration rate cell was analyzed by Image-J software.

At 48 h of treatment with 12.5 µg/mL of extracts, consistent with radical scavenging activity and cytotoxicity assay, the reflux technique possessed the highest proliferation rate (78.67% ± 4.85) followed by UAE (63.12% ± 3.69) and agitation (61.09% ± 7.27), respectively. The result was in agreement with other studies suggesting the pivotal role of antioxidants on wound recovery [35,36]. Excessive and prolonged reactive oxidant species during the inflammatory phase may damage the surrounding tissues and lead to the impairment of wound healing. Therefore, the choice of extraction technique may contribute to the conservation of these protective components associated with radical scavenging activity, hence stimulating the migration of cells toward the wound area. Representative images of the cell migration after 24 h and 48 h of treatment with extracts can be visualized in Figure 4.

#### 2.3.3. Collagen Synthesis by *C. papaya* Extracts

During the wound-healing process, an abundance secretion of collagen is stimulated and released to accelerate epithelial regeneration. As shown in Table 4, at 24 h post-treatment, HSF1184 cells synthesized a significant (*p* ≤ 0.05) number of collagen except when cells were treated with the UAE extraction technique. However, at 48 h post-treatment, collagen production rapidly increased to a greater level than at 24 h treatment. Additionally, collagen synthesis was more pronounced in cells treated with extract obtained from the reflux method as compared to agitation and the UAE method. This result could be related to the type of solvent used during the extraction process. As previously discussed, at any non-conventional extraction method applied, the extraction competence depends on the choice of the solvent. [28]. Methanol extract from *C. papaya* was postulated to be the most efficient solvent as compared to water, ethanol, methanol, n-butanol, dichloromethane, ethyl acetate, and n-hexane for antioxidant and antibacterial effects [13], and it is believed that these effects could contribute to the activities of wound healing. Moreover, these provide rational as to why old practitioners apply heat to extract the plant benefit. Moreover, the concentration used during this study was selected based on the migration assay, by which a concentration of 12.5 µg/mL promotes the highest migration activity on HSF1184 cells. The quantitative analysis of collagen stimulated by extracts was quantified using a calibration equation (y = 0.0136x − 0.0223, *R*^2^ = 0.9982). 

### 2.4. Metabolite Profiling of *C. papaya* Extract from Reflux Technique

The High Performance Liquid Chromatography Tandem Mass Spectrometry of Quadrupole Time-of-Flight (HPLC-MS QTOF) analysis of *C. papaya* from the reflux technique revealed a considerable amount of nine phytochemical compounds, including alkaloids and flavonoids (Table 5). HPLC chromatogram of these phytochemicals were illustrated in supplementary material (Figure S1). Data obtained from this analysis were in agreement with most reports describing that the most abundant compounds extracted from *C. papaya* were polar compounds [5,13]. A study has successfully isolated seven flavonoids from the butanol fraction of methanol extract from *C. papaya* leaves consisting of quercetin 3-(2^G^-rhamnosyrutinoside), kaempherol 3-(2^G^-rhamnosylrutinoside), quercetin 3-rutinoside, myricetin 3-rhamnoside, kaempherol 3-rutinoside, quercetin, and kaempherol, which have been tested on antioxidant activity by peroxynitrite scavenging assays [14]. In addition, methanol was found to extract many compounds rather than other solvents [37]. Flavonoids are well known for secondary metabolites that show a good significant contribution against wound-healing activities [38,39]. Kaempherol and luteolin are classified under flavonoid compounds that have been proved to exert wound-healing activities on diabetic and non-diabetic rats [38,39,40]. In the *C. papaya* plant, carpaine is a major alkaloid compound. It has been evaluated to exert antithrombocytopenic effects [19]. All these compounds supported that the synergistic effect of these phytochemical compounds present in the *C. papaya* leaves extract contributed to the bioactivities of *C. papaya*. 

## 3. Materials and Methods 

### 3.1. Materials 

2, 2-diphenyl-1-picrylhydrazyl (DPPH), thiazolyl blue tetrazolium bromide (MTT), and ascorbic acid were purchased from Sigma-Aldrich^®^ (St. Louis, MO, USA). HPLC-grade methanol, formic acid, ethanol, acetonitrile, and Folin–Ciocalteu reagent were purchased from Merck^®^ (Darmstadt, Germany). Penicillin streptomycin, Dulbecco’s modified eagle medium (DMEM), trypsin Ethylenediaminetetraacetic acid (EDTA), phosphate-buffered saline (PBS), and fetal bovine serum (FBS) were obtained from GIBCO^®^ (Carlsbad, CA, USA). Insoluble collagen assay-Sircol™ was purchased from Biocolor (Carrickfergus, UK). 

### 3.2. Plant Material Collection 

The green leaves of *C. papaya* cultivar “Eksotika” were collected from the Malaysian Agriculture Research and Development Institute (MARDI) (Serdang, Selangor, Malaysia). The sample was authenticated by botanist Dr. Shamsul Khamis, and a voucher specimen (SK 3143/17) was deposited at the Herbarium Institute of Bioscience, Universiti Putra Malaysia, Serdang, Selangor, Malaysia. Leaves were washed with tap water and dried in an oven at 50 °C for 2 days and then blended to obtain a fine powder using a Waring blender.

### 3.3. Extraction

#### 3.3.1. Ultrasonic-Assisted Extraction (UAE) 

UAE was performed using a Fisherbrand™ Q125 probe sonicator instrument. *C. papaya* powder (7.5 g) were extracted in 100 mL of methanol for 20 min at a frequency of 60 Hz. The extract was filtered using Whatman No. 1 filter paper and evaporated to dryness. The dried extracts were stored at −20 °C until further use. 

#### 3.3.2. Reflux

Reflux extraction was carried out by heating 7.5 g of round leaves of *C. papaya* in 100 mL of methanol for 20 min. The extract was filtered using Whatman No. 1 filter paper and evaporated to dryness. The dried extract was stored at −20 °C until further use. 

### 3.4. Agitation

The agitation method was carried out using an incubation shaker (Sartorius Certomat^®^ SII, Goettingen, Germany). A similar amount of *C. papaya* powder was extracted in 100 mL of methanol. The extract was agitated at 200 rpm for 20 min at room temperature. The extract was filtered using Whatman No. 1 filter paper and evaporated to dryness. The dried extract was stored at −20 °C until further use. 

### 3.5. Recovery Yield

The yield of *C. papaya* extract obtained from all extraction techniques was calculated based on Equation (1).

(1) $$Recovery\;Yield\;\left(\%\right)=\frac{W_{1}}{W_{0}}\times 100$$

where *W*_1_ is the weight of sample after drying, and *W*_0_ is the weight of dried leaves powder before drying.

### 3.6. Qualitative Secondary Metabolites Analysis

Extracts were subjected to qualitative secondary metabolites screening to identify the presence of saponins, flavonoids, terpenoids, steroids, coumarins, alkaloids, phenolics, and tannins. The analysis was carried out as described by Trease and Evans [41]. 

### 3.7. Test for Saponins

One mL of leaves extract was diluted with 3 mL of distilled water. Then, it was shaken for 15 min. The formation of a 1 cm layer of foam indicates the presence of saponin.

### 3.8. Test for Flavonoids

One mL of leaves extract was diluted with 1 mL of sodium hydroxide and hydrochloric acid. The development of yellow solution indicates the presence of flavonoids. 

### 3.9. Test for Terpenoids 

One mL of leaves extract was mixed with 2 mL of chloroform and 2 mL of concentrated sulfuric acid. The formation of a reddish-brown color at the interface indicates the presence of terpenoids.

### 3.10. Test for Steroids

One mL of leaves extract was mixed with 10 mL of chloroform followed by the addition of 10 mL of concentrated sulfuric acid. The formation of a red color on the upper layer indicates the presence of steroids while the addition of sulfuric acid showed yellow with green fluorescence.

### 3.11. Test for Coumarins

One mL of leaves extract was mixed with 1 mL of sodium hydroxide (10%). The yellow color indicates the presence of coumarins.

### 3.12. Test for Alkaloids

An equal volume of extracts was mixed with concentrated hydrochloric acid. Then, a few drops of Mayer’s reagent were added into a mixture. The presence of a green color or white precipitate indicates the presence of alkaloids.

### 3.13. Test for Phenolics and Tannins

One mL of leaves extract was mixed with 2 mL of distilled water followed by a few drops of ferric chloride (10%). A blue or green color indicates the presence of phenols.

### 3.14. Determination of 1,1-diphenyl-2-picrylhydrazy (DPPH) Scavenging Activity

The scavenging activity of extracts against DPPH radical was assessed based on the method suggested by Maisarah et al., with some modifications [42]. Prior to reaction, extracts were diluted in methanol to produce a series of concentrations from 0.065 to 0.5 mg/mL. Then, DPPH solution (0.2 mM) (100 µL) was added into each well plate containing different concentrations of the extracts. The mixture was kept in the dark at room temperature for 30 min. Ascorbic acid and quercetin were used as reference standards. The absorbance was measured at 517 nm, whereas the activity of radical scavenging was calculated using Equation (2). The values of inhibitory concentration (IC_50_) were determined using Graphpad Prism 5 software.

(2) $$DPPH\;scavenging\;activity\;\left(\%\right)=\frac{\left(A_{0}-A_{1}\right)}{A_{0}}\;\times 100$$

where *A*_0_ is the absorbance of the control and *A*_1_ is the absorbance of the extract or standard.

### 3.15. Cell Culture and Maintenance

Human skin fibroblast (HSF1184) cells (ECACC, United Kingdom) were maintained in DMEM, supplemented with 10% FBS and 1% Penicillin Streptomycin (PS), and incubated under 5% CO_2_ at 37 °C. Prior to the in vitro analysis, the extracts were prepared by dissolving in the cell culture media (without serum) and diluted to a respective concentration applied.

### 3.16. Cytotoxicity Assay via Sulforhodamine B (SRB) Assay

Cytotoxicity assay on HSF1184 was conducted according to Vinchai and Kirtikara, with modifications [43]. Briefly, 15,000 cells were seeded in 96-well culture plates and incubated overnight. Cells were treated with extracts (12.5 µg/mL to 500 µg/mL) for 24 h. Cells’ viability was assayed by SRB. Color development was measured at 510 nm.

### 3.17. Scratch Assay

A scratch assay was carried out according to Ahmad et al., with minor modifications [44]. Briefly, 300,000 cells were seeded into six-well plates and allowed to reach confluency for 48 h. A linear scratch was made using a sterile fine tip and rinsed with PBS to remove cell debris. Subsequently, 3 mL of the extracts ranging from 12.5 µg/mL to 100 µg/mL was added into each well. Ascorbic acid (5 ug/mL) was served as positive control, while cell culture media without serum served as the negative control. The closure of the scratch area was recorded after 48 h of treatment using an inverted microscope equipped with a digital camera. The image was analyzed using image analysis software, Image J. The percentage of closure was calculated using Equation (3).

(3) $$Migration\;rate\;\left(\%\right)=\;\frac{Area\;t:0-Area\;t:48}{Area\;t:0}\;\times 100$$

where *t*: 0 is the average scratch area at ‘0’ time, and *t*: 48 is the average scratch area after 48 h of treatment.

### 3.18. Collagen Synthesis Assay 

Collagen syntheses by cells were measured using Sircol collagen assay (Biocolor Ltd, Carrickfergus, UK). Pre-confluent cells were treated with 12.5 µg/mL of extracts for 48 h. Spent media was collected and mixed with 200 µL of isolation and concentration reagent. The mixture was incubated overnight at 4 °C. One mL of Sircol dye reagent was added and mixed vigorously for 5 min. Then, the mixture was centrifuged at 12,000 rpm for 10 min, and the supernatant was removed prior to the addition of 750 µL ice-cold acid–salt wash reagent. After centrifugation and removal of the supernatant, an alkali reagent was added into the tube and the mixture was measured at 555 nm.

### 3.19. HPLC-MS/MS-QTOF Analysis

Metabolite profiling analysis was performed on an Agilent 6560 Ion Mobility connected to a Quadrupole Time-of-Flight (IM-QTOF) HPLC/MS system equipped with a dual electroscopy ionization (ESI) source (Agilent Technologies, Santa Clara, CA, USA). Chromatographic separation was achieved on a Zorbax Eclipse plus C18 column (2.1 mm × 50 mm, 1.8 µm) (Agilent Technologies, Santa Clara, CA, USA) operated at 25 °C employing a gradient elution using 0.1% formic acid in water [A] and 0.1% formic acid in methanol [B] as the mobile phase at a flow rate of 0.3 mL/min. The following gradient elution was adopted: 35−90% B for the first 0 to 7 min; 90−90% B for 7 to 25 min; 90−35% B for 25 to 35 min; the initial condition was maintained for 5 min. The sample injection volume was 3 μL.

The mass spectrometer was operated in both positive and negative electrospray ionization modes. The mass spectra were recorded by scanning the mass range from *m*/*z* 100 to 3000 in both MS and MS/MS modes. The gas temperature was set to 200 °C, and the drying gas flow rate was 12 L/min. The nebulizer pressure was 20 psi, the sheath gas temperature was 400 °C, and the sheath gas flow was 12 L/min. The source parameters such as capillary voltage was 2500 V, whilst the nozzle voltage was 500 V.

Data analysis was performed using Agilent MassHunter Qualitative software, Version B.06.00 (Agilent Technologies, Santa Clara, CA, USA) with Molecular Feature Extractor (MFE) algorithms in concert with Mass Profiler Professional software, Version 12.1 (Agilent Technologies, Santa Clara, CA, USA). The compound identification was carried out by using a Personal Compound Database Library (PCDL) (Agilent, Santa Clara, CA, USA) with the METLIN Personal Metabolite Database and a customized PCDL database (peptides, metabolites, lipids, AMRT, and AM).

### 3.20. Statistical Analysis

All data were analyzed by using one-way ANOVA, SPSS Version 17.0 (IBM corporation, New York, U.S.A). The significance of the results was determined by Tukey’s test, and a *p* value of 0.05 was set as the limit of significant difference. The results were expressed as mean ± standard deviation (STDEV) or standard error mean (SEM).

## 4. Conclusions

Our finding concluded that the extraction of *C. papaya* was optimum using the reflux technique, which produced the highest antioxidant activity, promoted cells proliferation and migration, and was an excellent promoter for collagen synthesis as well. Further studies should explore the optimum conditions of the reflux process for the wound-healing properties of methanolic extracts of *C. papaya.*

## Figures and Tables

**Table 1:** Extraction yield of *C. papaya* leaves obtained by different extraction techniques. UAE: ultrasonic-assisted extraction.

Extraction Techniques	Extraction Yields (%)
UAE	13.57 ± 0.18 ^a^
Reflux	17.86 ± 1.61 ^b^
Agitation	15.86 ± 0.91 ^c^

Values are presented as mean ± standard deviation of three replicates. The different letter indicates there are significant differences (*p* ≤ 0.05).

**Table 2:** Secondary metabolites of *C. papaya* leaves extract.

Test	UAE	Reflux	Agitation
Saponins	+	+	+
Flavonoids	+	+	+
Terpenoids	-	-	-
Steroids	-	-	-
Coumarins	+	+	+
Alkaloids	+	+	+
Phenolics	+	+	+

–: indicates Absence, +: indicates Presence.

**Table 3:** IC_50_ of 2, 2-diphenyl-1-picrylhydrazyl (DPPH) radical scavenging activity of *C. papaya* leaves extracts.

Sample	IC_50_ (mg/mL)
**Extracts**	
UAE	0.377 ± 0.014 ^a^
Reflux	0.236 ± 0.009 ^b^
Agitation	0.404 ± 0.009 ^c^
**Positive Control**	
Ascorbic acid	0.014 ± 0.002 ^d^
Quercetin	0.00625 ± 0.001 ^e^

Data represent the mean ± SD of three independent experiments. The different letters indicate that there are significant differences (*p* ≤ 0.05).

**Table 4:** Effects of *C. papaya* extract treatment from various extraction techniques on collagen synthesis in HSF1184 cells after 24 and 48 h at a concentration of 12.5 µg/mL.

Extracts	24 h	S.E.M	48 h	S.E.M
UAE	100.59	5.28	136.31 *	0.39
Reflux	131.65 *	5.86	164.89 *	0.67
Agitation	119.93 *	4.69	160.25 *	5.22
Control	100	-	100	-

* *p* ≤ 0.05.

**Table 5:** Mass spectral analysis of the compounds present in the extract of *C. papaya* leaves using the reflux method.

No.	*t*_R_ (min)	Compound	Mass	Difference (ppm)	Score
1.	1.64	Carpaine	478.379	−3.66	98.48
2.	1.807	Kaempferol 3-(2G-glucosylrutinoside)	756.2128	−1.97	96.85
3.	2.069	Kaempferol 3-(2″-rhamnosylgalactoside) 7-rhamnoside	740.2185	−2.88	97.22
4.	2.14	Kaempferol 3-rhamnosyl-(1->2)-galactoside-7-rhamnoside	740.2187	−3.07	96.85
5.	2.447	Luteolin 7-galactosyl-(1->6)-galactoside	610.1560	−4.35	97.55
6.	2.951	Orientin 7-O-rhamnoside	594.1606	−3.67	98.19
7.	4.913	11-hydroperoxy-12,13-epoxy-9-octadecenoic acid	328.2263	−3.99	98.95
8.	5.386	Palmitic amide	255.257	−4.18	94.74
9.	6.632	2-Hexaprenyl-6-methoxyphenol	532.428	−0.31	98.13

*t*_R_, retention time.

## Supplementary Materials

The following are available online at https://www.mdpi.com/1420-3049/25/3/517/s1, Figure S1: Compounds profiles of *C. papaya* obtained from reflux technique.
