# Supplementary figures and images for: Bioassay-Guided Different Extraction Techniques of Carica papaya (Linn.) Leaves on In Vitro Wound-Healing Activities
Source: Molecules. 2020 Jan 24;25(3):517. doi: 10.3390/molecules25030517 (PMC7037417; doi:10.3390/molecules25030517)

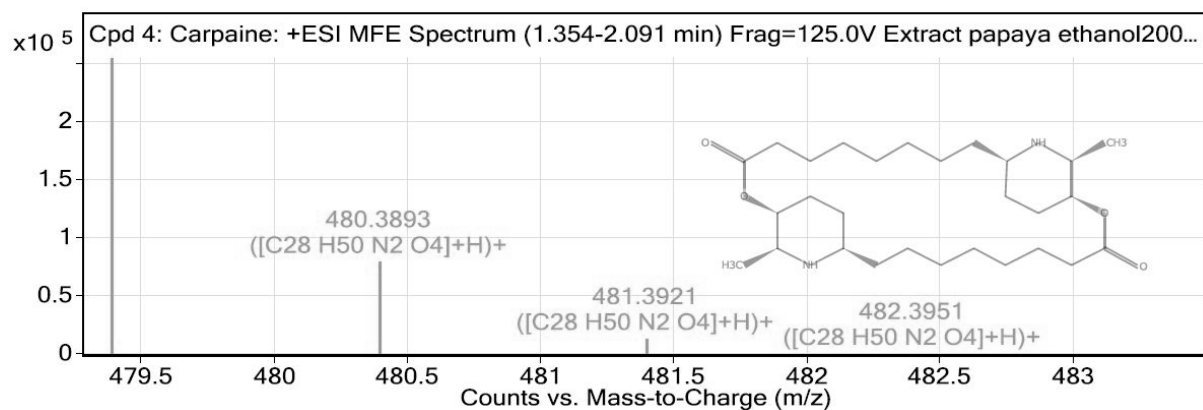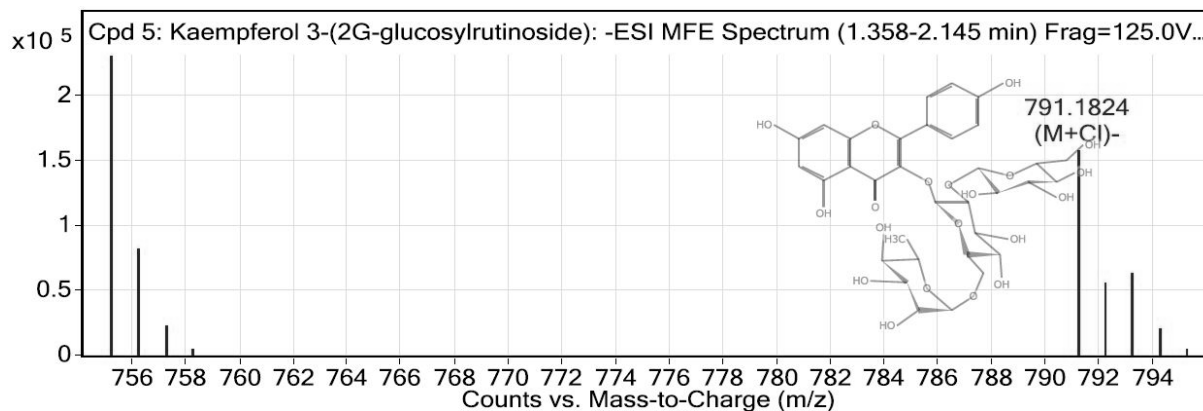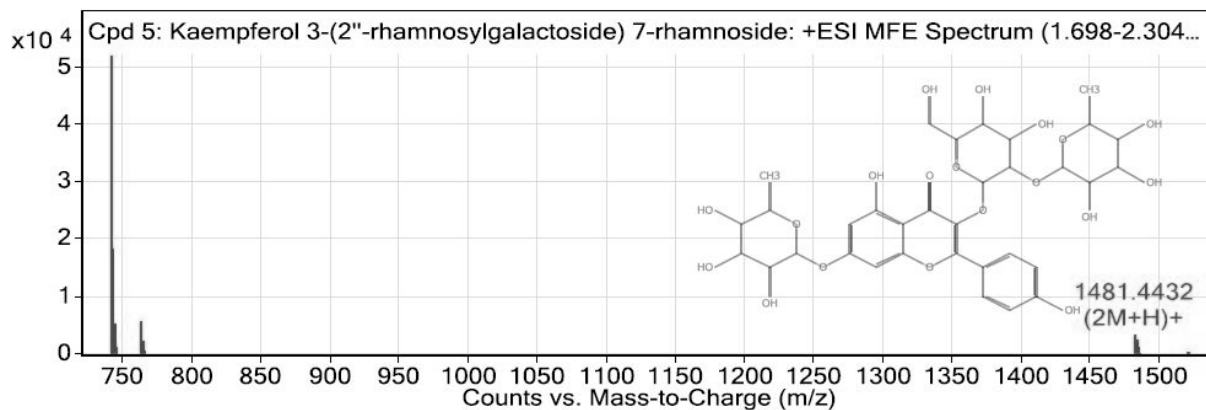

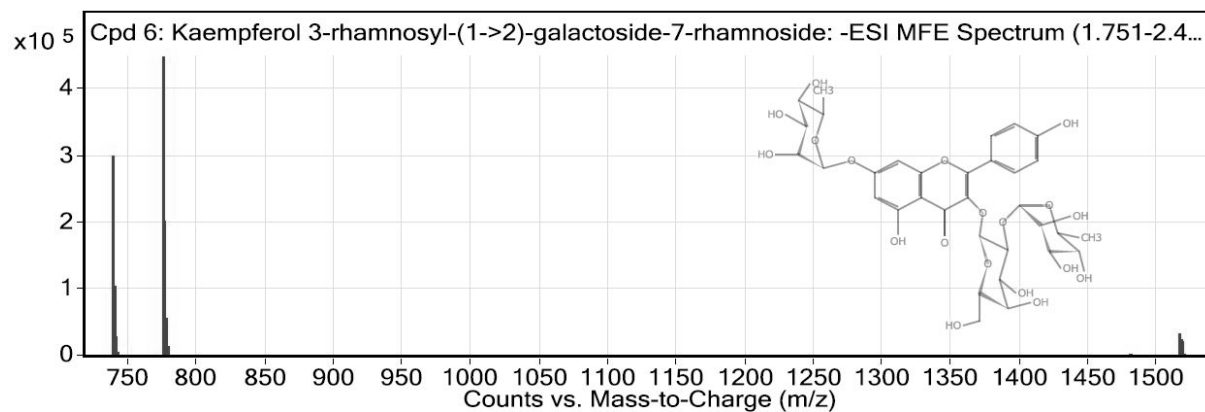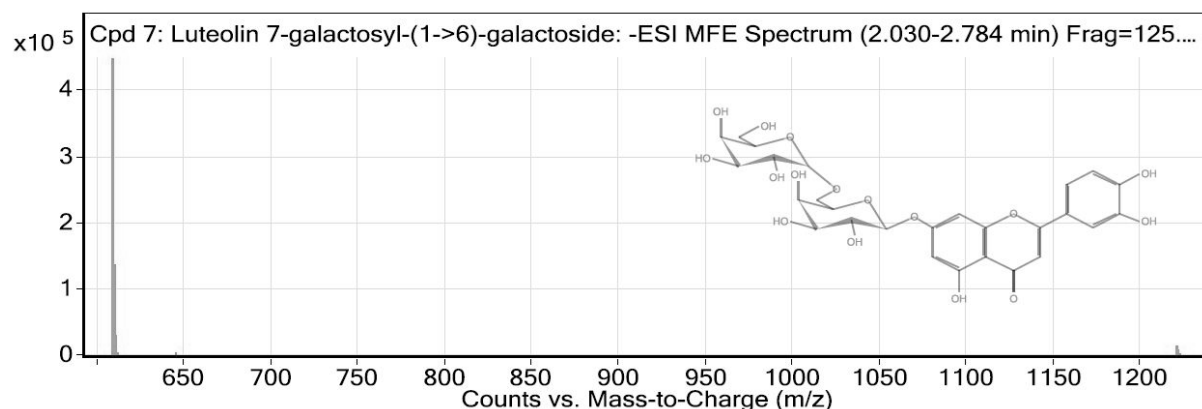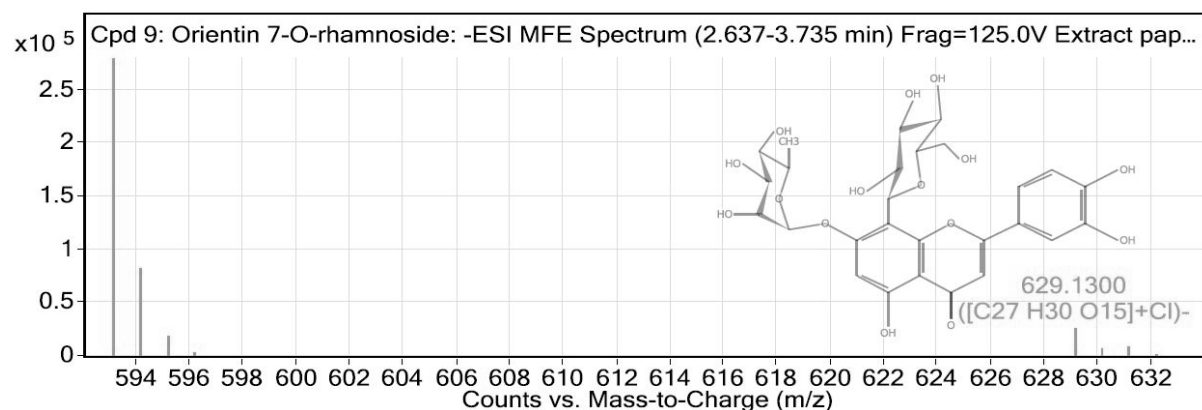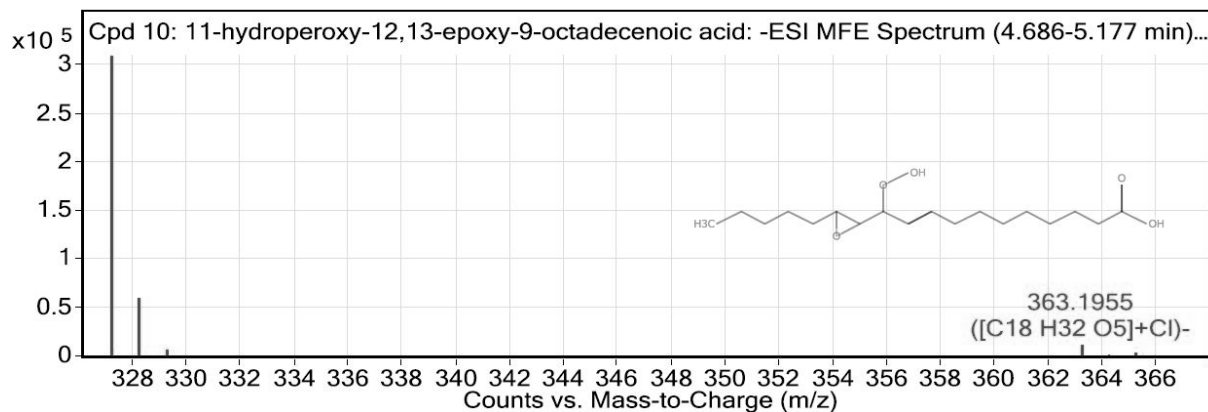

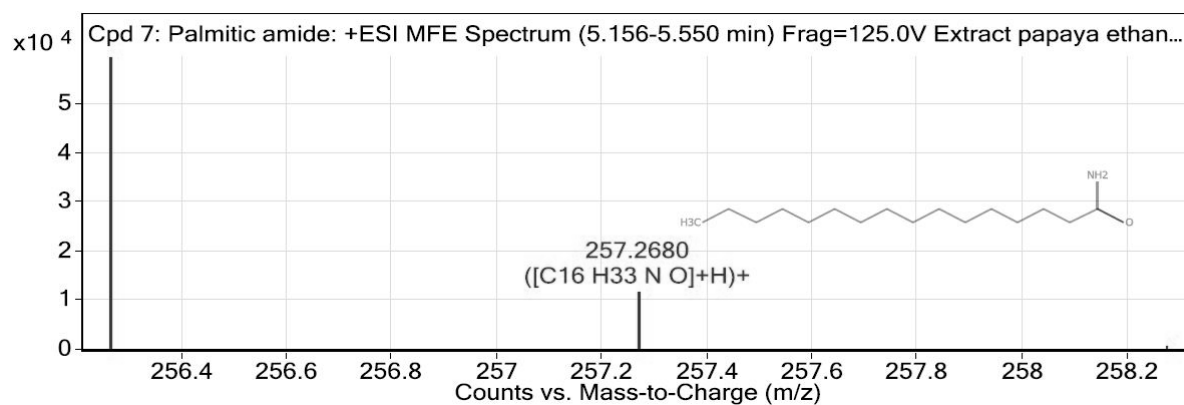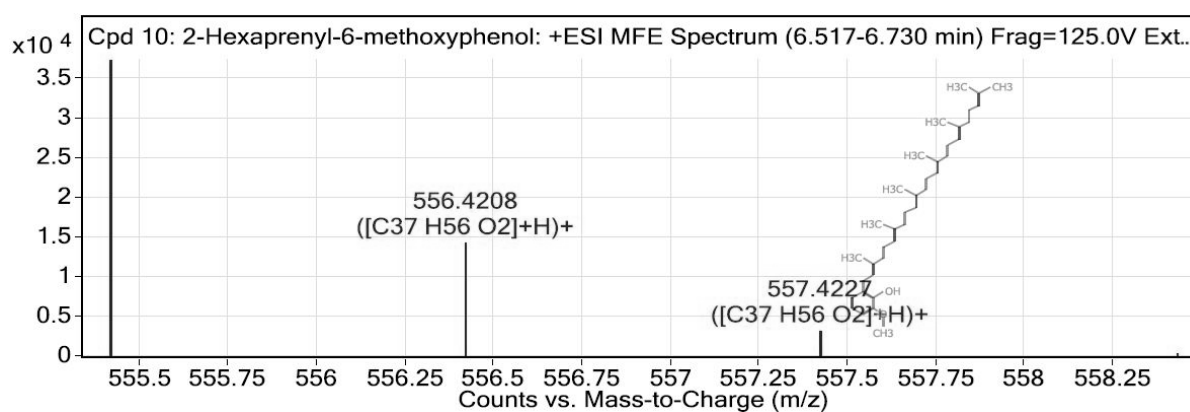

**Figure S1.** Compounds profiles of *C. papaya* obtained from reflux technique.

Supplement: Supplementary file 1 [file molecules-25-00517-s001.pdf]
